# Diagnosing celiac disease by video capsule endoscopy (VCE) when esophogastroduodenoscopy (EGD) and biopsy is unable to provide a diagnosis: a case series

**DOI:** 10.1186/1471-230X-12-90

**Published:** 2012-07-19

**Authors:** Matthew S Chang, Moshe Rubin, Suzanne K Lewis, Peter H Green

**Affiliations:** 1 Celiac Disease Center, Division of Digestive and Liver Diseases, Department of Medicine, Columbia University College of Physicians and Surgeons, New York, NY, USA; 2 Division of Gastroenterology and Hepatology, Department of Medicine, New York Hospital Queens, Weill Cornell Medical College, Flushing, NY, USA

**Keywords:** Gluten-free diet, Duodenum, Small intestine, Diagnostic techniques, Contraindications

## Abstract

**Background:**

Video capsule endoscopy (VCE) is mainly used to evaluate patients with celiac disease in whom their course after diagnosis has been unfavorable and the diagnosis of adenocarcinoma, lymphoma or refractory celiac disease is entertained, but it has been suggested that VCE could replace esophagogastroduodenoscopy (EGD) and biopsy under certain circumstances.

**Methods:**

We report a single center case series of 8 patients with suspected celiac disease who were diagnosed by VCE.

**Results:**

EGD and biopsy had been performed in 4 patients resulting in a negative biopsy, declined by 2, and contraindicated in 2 due to hemophilia and von Willebrand disease. In all patients, mucosal changes of scalloping, mucosal mosaicism and reduced folds were seen in either the duodenum or jejunum on VCE. Follow-up in 7 patients demonstrated improvement in either their serological abnormalities or their presenting clinical features on a gluten-free diet.

**Conclusions:**

Our case series demonstrates that VCE and the visualization of the characteristic mucosal changes of villous atrophy may replace biopsy as the mode of diagnosis when EGD is either declined or contraindicated, or when duodenal biopsies are negative and there remains a high index of suspicion. Further study is needed to clarify the role and cost of diagnosing celiac disease with VCE.

## Background

Esophagogastroduodenoscopy (EGD) with duodenal biopsy is considered the gold standard for the diagnosis of celiac disease. However, the histological lesions characteristic for celiac disease may be missed in cases of patchy duodenal atrophy, even with multiple duodenal biopsies [1,2]. Despite this approach, some patients with a clinical presentation that is highly suggestive for celiac disease may still have a normal appearing EGD and non-diagnostic biopsy. These patients are usually not placed on a gluten-free diet because of the lack of pathological confirmation of villous atrophy. In addition some patients may not be candidates for EGD because of relative medical contraindications, such as from a bleeding diathesis, or fear of the procedure.

Video capsule endoscopy (VCE) provides high-resolution magnified views of the small intestinal mucosa in a noninvasive manner and has been shown to be sensitive (76–99%) and specific (56–100%) for identifying celiac disease [3]. Some features that can be observed with VCE include reduced duodenal folds; scalloping, layering, or stacking of folds; mucosal fissures, crevices, grooves, nodularity or a mosaic pattern [4]. Currently, VCE is mainly used to evaluate patients with celiac disease in whom their course after diagnosis has been unfavorable and the diagnosis of adenocarcinoma, lymphoma or refractory celiac disease is entertained [5]. VCE allows visualization of the entire small bowel, potentially locating more distal and patchy disease that may be missed by EGD [6]. Because of the high specificity for the presence of villous atrophy when the appropriate mucosal abnormalities are visualized, it has been proposed that VCE may replace EGD with biopsy in selected circumstances [5]. These include patients in whom there is a high clinical suspicion (supportive history, positive serologies), but a normal EGD and unremarkable biopsy and in those patients with bleeding diatheses and severe cardiopulmonary disease, or who decline EGD [5]. There has, however, been no literature supporting this approach. We therefore report a case series confirming the appropriateness of this method.

## Methods

### Patients

This was a retrospective review of eight patients seen at the Celiac Disease Center at Columbia University Medical Center (CUMC) for an evaluation of possible celiac disease. The Celiac Disease Center is a tertiary referral center that has a cohort of 1,285 patients with celiac disease. Patients that were included in our evaluation had both: 1) suspected celiac disease and 2) either a normal EGD with a non-diagnostic biopsy, or were unable to undergo EGD with biopsy, either because of medical co-morbidities or personal preference.

Patients were considered to have suspected celiac disease if their clinical presentation included the presence of at least one of the following: abdominal pain, chronic diarrhea, unexplained anemia, osteoporosis, unexplained neuropathy, and/or unexplained weight loss. Patients also had a positive serologic test, preferably either a positive anti-endomysial antibody (EMA) or anti-tissue transglutaminase (tTG) antibody. Patients were not on a gluten-free diet at the time of evaluation. A normal EGD and non-diagnostic biopsy was defined as having a Marsh score of 0. Patients that were unable to undergo biopsy for medical reasons primarily included patients at risk of excessive bleeding. Patients that declined EGD were also included. This study was reviewed and approved by the CUMC Institutional Review Board.

### Procedures

Patients underwent EGD at the CUMC Endoscopy Unit. At least 4–8 duodenal biopsies were performed as part of standard clinical care. Specimens were interpreted by a staff gastrointestinal pathologist. VCE was performed using the Given PillCamSB (Given Imaging, Yoqneam, Israel). VCE findings were considered as consistent with celiac disease if at least one of the following was present: reduced duodenal folds; scalloping, or reduction of folds; mucosal fissures, crevices, grooves, or a mosaic pattern [4]. VCE images were interpreted by gastroenterologists experienced in VCE. Localization within the small bowel was approximated using anatomic landmarks (major ampulla) for the duodenum and dividing the duration of the small bowel transit into two parts for the jejunum and ileum.

## Results

Eight patients were found to fulfill the criteria for suspected celiac disease and had undergone VCE confirming celiac disease (Table 1). Half were female with a median age of 25 (range 16–65 years). Patients underwent evaluation for celiac disease because of gastrointestinal complaints and diarrhea (n = 3), iron deficiency anemia (n = 2), osteoporosis (n = 1), neuropathy (n = 1), and for screening in the setting of a family history of celiac disease (n = 1). Six patients (75%) had serologic testing that was suggestive of celiac disease, two-thirds of which were either EMA or tTG positive, and two patients had a first-degree relative with celiac disease.

**Table 1:** Characteristics of patients who were diagnosed with celiac disease using video capsule endoscopy (VCE)

**Pt No.**	**Sex/Age**	**Indication for evaluation of celiac disease**	**Family History**	**Antibody status**	**EGD**	**Biopsy**
1	F/29	Osteoporosis	None*	+EMA	Normal	Normal
2	F/65	Peripheral neuropathy	None*	+AGA	Normal	Normal
3	F/20	Iron deficiency anemia	Sister	Negative	Normal	Normal
4	M/64	Diarrhea	None*	Negative	Normal	Normal
5	M/45	Diarrhea	None	+AGA	Declined	Not performed
6	M/58	Diarrhea	None	+EMA, TTG	Declined	Not performed
7	M/19	Screening for celiac disease due to family history	Father	+EMA, TTG	Not performed	Contraindicated - Hemophilia
8	F/16	Iron deficiency anemia	None†	+EMA, TTG	Not performed	Contraindicated - Von Willebrand disease

Abbreviations: anti-endomysial antibody (EMA), anti-gliadin antibody (AGA), anti-tissue transglutaminase antibody (TTG).

Immunoglobulins measured for AGA and TTG were IgG and IgA, respectively.

HLA typing when performed were denoted by * for DQ2 positive and † for DQ8 positive.

**Figure 1:**
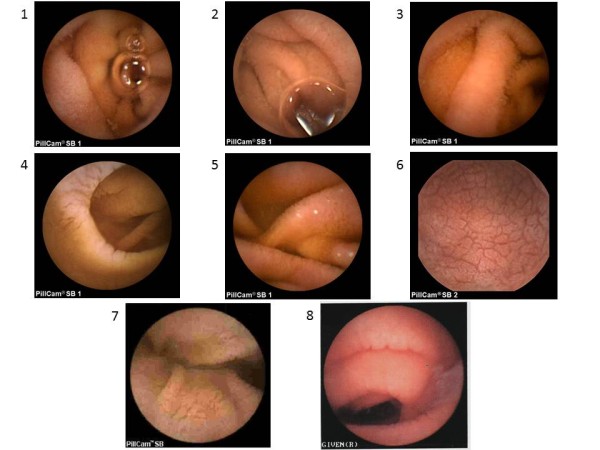
**Video capsule endoscopy images found in celiac disease according to patient.** Patient 1. Villous atrophy (absent villi), Patient 2. Villous atrophy (absent villi) and mild scalloping, Patient 3. Absent and reduced villi. Patient 4, Scalloping, crevices, atrophy, Patient 5. Absent jejunal villi, Patient 6. Mosaic, cobblestoned pattern with marked scalloping of folds, Patient 7. Crevices, atrophy, scalloping, Patient 8. Scalloping.

Four patients (50%) had a normal EGD and Marsh stage 0 biopsy. The other four patients never underwent EGD, of which two patients (patients 7 and 8) had a bleeding diathesis (von Willebrand disease and hemophilia) and two patients (patients 5 and 6) declined. Patient 5 reported a prior diagnosis of celiac disease as a child and did not have any records to confirm this, but preferred not to undergo EGD. Patient 6 declined EGD because of concerns regarding the potential associated complications. Mucosal changes suggestive of villous atrophy were seen in all patients by VCE in either the duodenum or jejunum, including scalloping, absent folds, or mucosal mosaicism (Figure 1). Mucosal erosions were identified in 4 patients. The capsule did not cross the ileocecal valve in patient 2, but a follow-up radiograph did not show a retained capsule and there were no complications from VCE.

All patients were seen by a trained dietitian and commenced a gluten-free diet; seven patients (88%) demonstrated improvement in either their serological abnormalities or their presenting clinical features on a gluten-free diet. Patients 4, 5, and 6 had improvement in diarrhea, patients 3 and 8 had improvements in anemia, and patient 1 had an improvement in her DEXA scan. This improvement was particularly dramatic for patient 3 who, prior to a gluten-free diet, had required transfusions and had been on both iron supplementation and oral contraceptives to control her menses. Patient 2 did not have any improvement in her neuropathy and gastrointestinal symptoms after 15 months on a gluten-free diet and was subsequently lost to follow up.

## Discussion

Our study demonstrates that VCE can be used to diagnose celiac disease in patients with suspected celiac disease who have either a non-diagnostic EGD with biopsy or who are unable or unwilling to undergo EGD. Although VCE is used to diagnose celiac disease in clinical practice, previous guidelines have not recommended this approach and our study is the first to support its role in diagnosing celiac disease. It is unclear what proportion of patients that have positive celiac disease serologies, but a normal EGD (these patients may be classified as having latent celiac disease and are usually not advised to commence a gluten-free diet), will have a VCE that is diagnostic for celiac disease. One small series did not find celiac disease in this situation [7], but there is growing evidence that celiac disease may sometimes vary in distribution, appearing more distally and patchy in nature [6,8]. However, before embarking on additional testing with VCE, it is important to first confirm the adequacy of the initial diagnostic work up, starting from a meticulous endoscopic evaluation, to taking a sufficient number biopsies, and finally having an experienced gastrointestinal pathologist interpret the pathology. Additionally, a trial of a gluten-free diet as a diagnostic method should be avoided as it can negatively impact quality of life, is difficult to adhere to, and overall more expensive, particularly in the United States where there is limited availability of gluten-free foods.

Traditionally celiac disease is diagnosed by biopsy of the duodenum in individuals with positive serological tests. Negative biopsies in this setting result in a consideration that the serological tests were false positives, or the patient has potential or latent celiac disease. This result could however be the result of either an inadequate number of biopsies [2] or that the duodenal bulb was not biopsied [9]. Our results would suggest that when the index of suspicion is high, VCE may confirm the diagnosis of celiac disease in this setting. This is based on the high specificity for the mucosal abnormalities associated with the presence of villous atrophy. However, a negative VCE in which the characteristic endoscopic appearance of villous atrophy is not appreciated does not exclude celiac disease, as villous atrophy may be present in the setting of a normal endoscopic appearance (low sensitivity).

Our findings were limited by our small cohort size and retrospective study design. Ideally we would have also confirmed mucosal recovery in addition to symptomatic and laboratory improvement with a repeat VCE while on a gluten-free diet. Although we were unable to obtain a tissue diagnosis of celiac disease in our series, the vast majority of the patients developed clinical improvement after starting a gluten-free diet, which was able to confirm the diagnosis of celiac disease. Patients with suspected celiac disease that do not improve on a gluten-free diet should be followed closely by a nutritionist experienced with celiac disease, and may possibly benefit from a repeat VCE with or without a gluten challenge.

## Conclusion

In our selected series of patients the presence of villous atrophy was confirmed by the VCE appearance of the duodenal or jejunal mucosa. While the sensitivity of diagnosing celiac disease by VCE in the presence of severe degrees of villous atrophy appears high [8], it remains to be seen whether newly developed computerized image analysis techniques can facilitate the detection of lesser degrees of villous atrophy [10]. Larger, controlled trials are needed to confirm the accuracy of VCE as compared to EGD with biopsy in diagnosing, rather than monitoring, celiac disease. Further studies documenting the cost effectiveness of VCE as compared to EGD, and patient preference studies, need to be performed.

## Abbreviations

(EGD) = Esophagogastroduodenoscopy; (VCE) = Video capsule endoscopy; (CUMC) = Columbia university medical center; (EMA) = Anti-endomysial antibody; (tTG) = Anti-tissue transglutaminase.

## Competing interests

Dr. Green has relationships with Alvine (consultant, advisory committee), Alba Therapeutics (advisory committee). Dr. Rubin is a consultant for Given Imaging. Dr. Chang and Dr. Lewis have no potential competing interests.

## Authors’ contributions

MC and PG were responsible for the study concept, design, data analysis, and drafted the initial manuscript. MC, MR, SL, PG were involved in manuscript review and revision. All authors read and approved the final manuscript.

## Pre-publication history

The pre-publication history for this paper can be accessed here:

http://www.biomedcentral.com/1471-230X/12/90/prepub
